# A Very Desirable Hypnotic

**Published:** 1911-06

**Authors:** 


### ABSTRACTS

---

### A Very Desirable Hypnotic

In the discussion following the reading of a paper entitled "Acute poisoning from trional.—A clinical and anatomic pathological study," by Drs. Gaultier, Caillaud and Tomovici before the Société de Therapeutique, Paris, November 23, 1910, Prof. Bardet stated in part as follows:

"The case described by the authors presents marked medullary phenomena such as were a common occurrence with the since abondoned chloralose, are not infrequently reported following the use of sulfonal and have even been charged to diethyl-barbituric acid, known as veronal. The latter is practically insoluble and only very slowly absorbed. One would therefore expect it to be of low toxicity; nevertheless its action is very insidious and frequently unduly prolonged with disagreeable and sometimes serious by-effects.

Medinal, which is the sodium salt of this acid, on the other hand, is readily soluble, very rapidly absorbed and to my knowledge has so far never caused accidents, even when administered subcutaneously. It is a very reliable hypnotic, devoid of unduly protracted action and renders excellent services."

